# Supplementary material for: The Mantle Fe3+/ΣFe Ratio Has Doubled Since the Early Archean
Source: Nat Commun. 2026 Jan 14;17:429. doi: 10.1038/s41467-025-66969-1 (PMC12804952; doi:10.1038/s41467-025-66969-1)
Supplement: Supplementary file 1 — Supplementary Information [file 41467_2025_66969_MOESM1_ESM.pdf]

Supplementary Information for

The Mantle $Fe^{3+}/\Sigma Fe$ Ratio Has Doubled Since the Early Archean

Xiao-Xi Zhu[1,2], Wen-Yong Duan[1,3*], Taras Gerya[1], Xin Zhou[1], Jia-Cheng Tian[1]

[1] *Department of Earth and Planetary Sciences, Swiss Federal Institute of Technology, Zurich 8092, Switzerland*

[2] *State Key Laboratory of Tropical Oceanography, South China Sea Institute of Oceanology, Chinese Academy of Science, Guangzhou 510301, China*

[3] *Institute of Geology, Mineralogy and Geophysics, Faculty of Geosciences, Ruhr-University Bochum, Bochum 44780, Germany*

[*]Corresponding author: Dr. Wen-Yong Duan (wenyong.duan@rub.de).

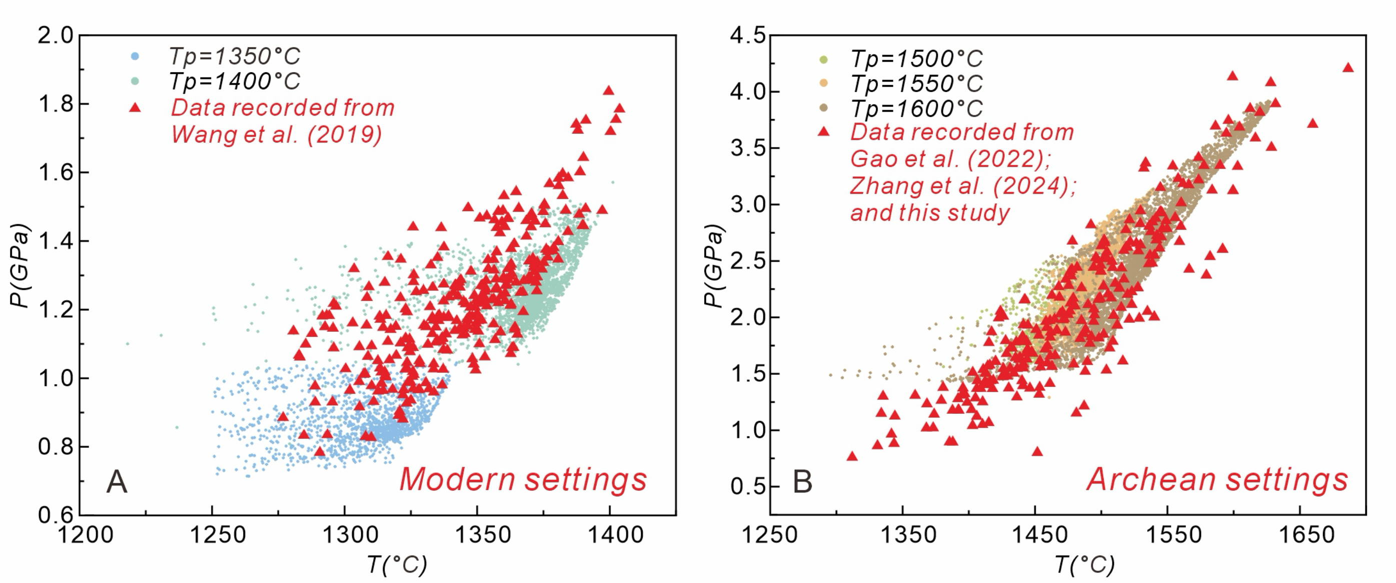

Fig. S1 *P*-*T* conditions of the reference model (circles; see Fig. 2) and of actual rock records used in this study and in previous works[1-3]. (A) Simulation results under plausible modern mantle potential temperatures (*Tp*), alongside the *P*-*T* conditions recorded by modern MORB samples; (B) Simulation results under plausible Archean mantle *Tp* conditions, alongside the *P*-*T* conditions recorded by Archean MORB-like samples.

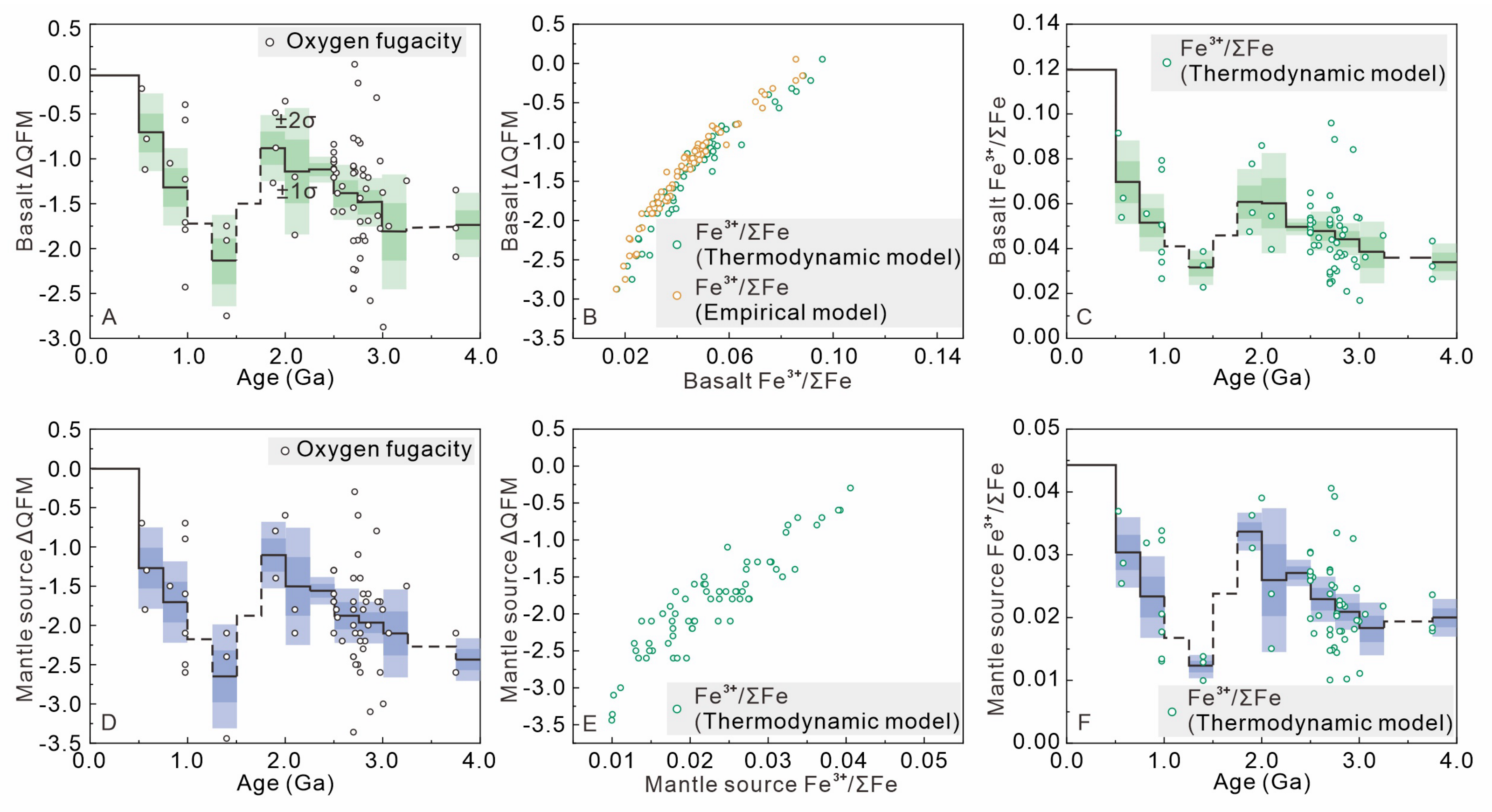

Fig. S2 Changes of oxygen fugacity and $Fe^{3+}/\Sigma Fe$ ratio in MORB-like basalts and mantle source over time. (A) the oxygen fugacity of basalt varying over time using *P*-*T*-V/Ti conditions of MORB-like basalts[1,2]; (B) whole rock $Fe^{3+}/\Sigma Fe$ of basalt (thermodynamic and empirical methods) corresponding to different *P*-*T*-fO_2 conditions and whole-rock major elements of MORB-like basalts; (C) the whole rock $Fe^{3+}/\Sigma Fe$ of basalt varying over time using thermodynamic methods; (D) the oxygen fugacity of mantle varying over time using *P*-*T*-$Fe^{3+}/\Sigma Fe$ conditions of basalts; (E) whole rock $Fe^{3+}/\Sigma Fe$ of mantle corresponding to different *P*-*T*-$Fe^{3+}/\Sigma Fe$ conditions of basalts (thermodynamic and empirical method); (F) the whole rock $Fe^{3+}/\Sigma Fe$ of mantle varying over time.

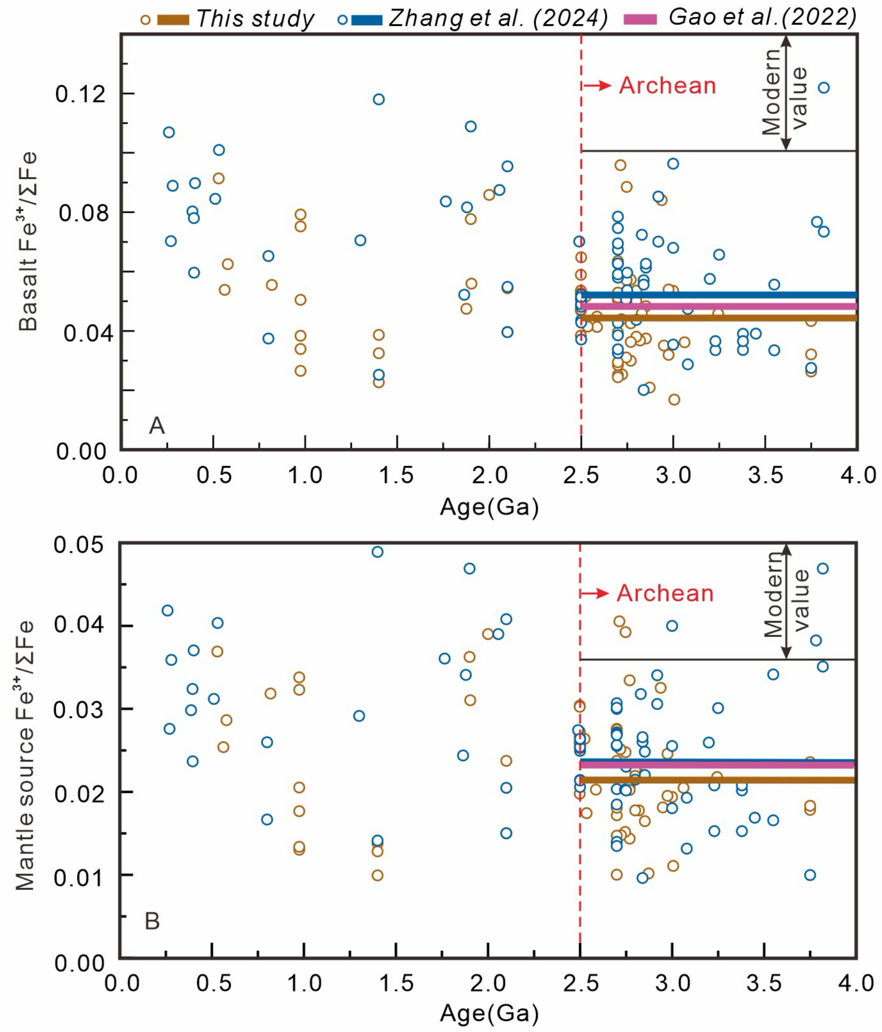

Fig. S3 The whole rock $Fe^{3+}/\Sigma Fe$ in MORB-like rocks and mantle calculated in three databases (in the references listed on top of panel A). Different colored lines represent the Archean average value from three databases. (A) The whole rock $Fe^{3+}/\Sigma Fe$ in MORB-like rocks based on P-T-fO_2 conditions of MORB-like rocks; (B) The whole rock $Fe^{3+}/\Sigma Fe$ in mantle sources based on P-T-$Fe^{3+}/\Sigma Fe$ conditions of MORB-like rocks.

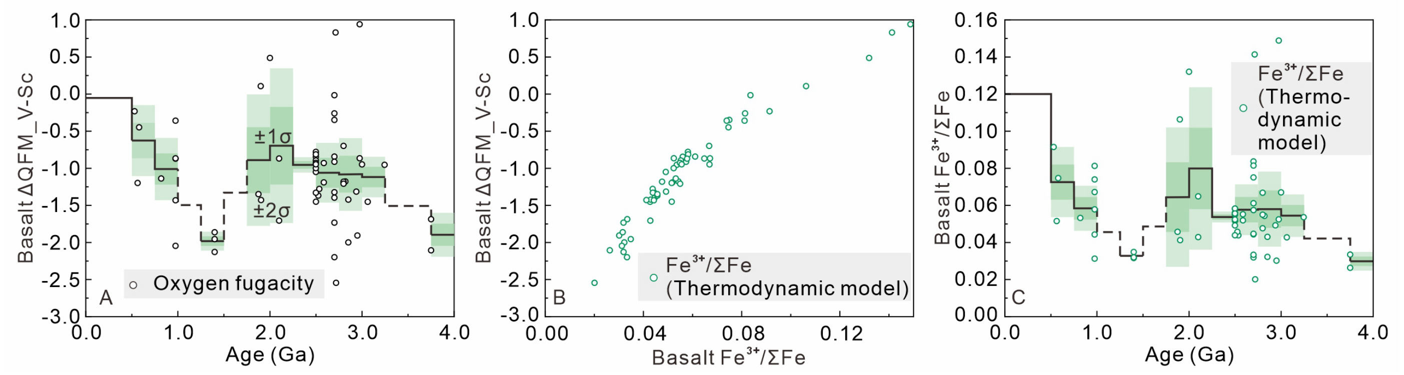

Fig. S4 Record results of V-Sc oxybarometer[1,2] and corresponding whole rock $Fe^{3+}/\Sigma Fe$ of MORB-like basalt. (A) the oxygen fugacity of basalt varying over time using *P*-*T*-V/Sc conditions of MORB-like basalts[1,2]; (B) whole rock $Fe^{3+}/\Sigma Fe$ of basalt (thermodynamic methods) corresponding to different P-T-fO_2 conditions and whole-rock major elements of MORB-like basalts; (C) the whole rock $Fe^{3+}/\Sigma Fe$ of basalt varying over time using thermodynamic methods.

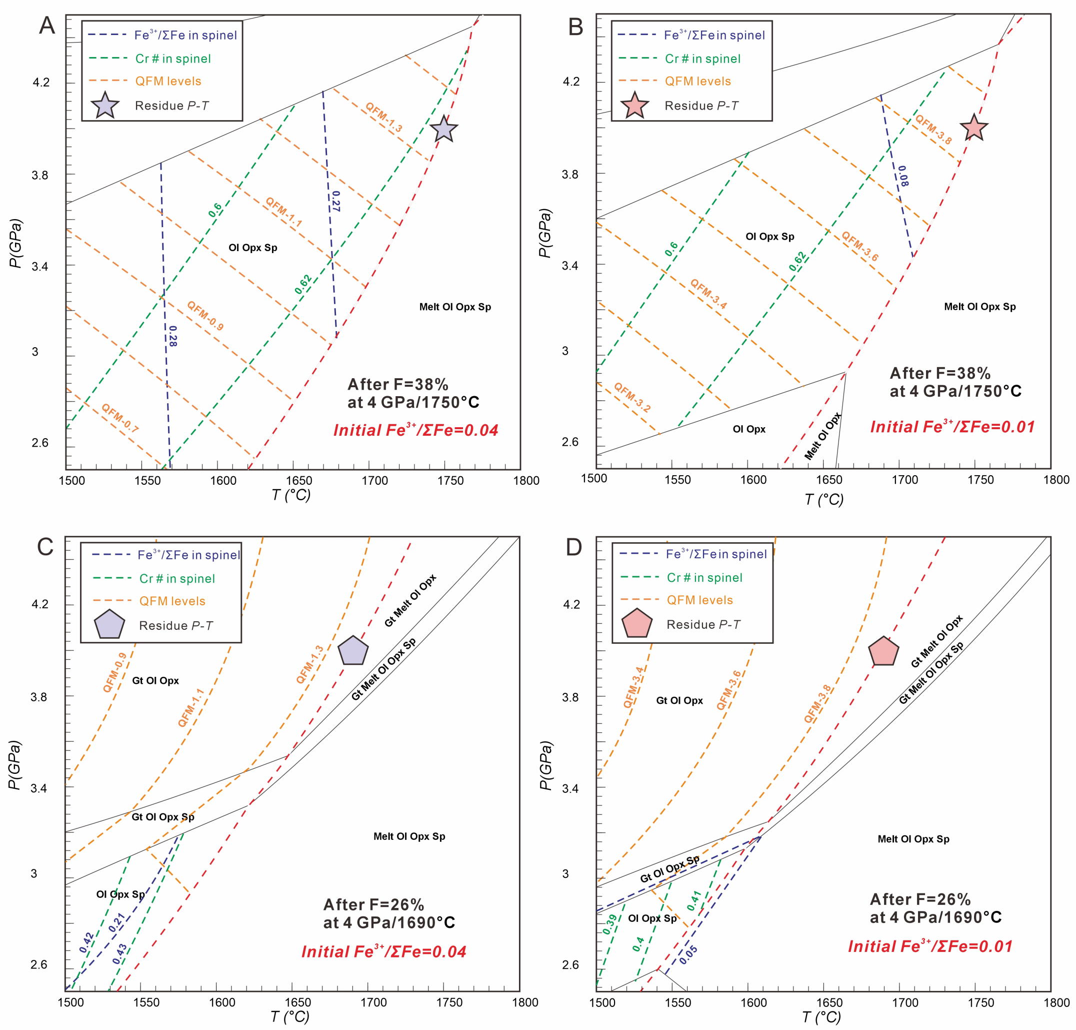

Fig. S5 Phase diagram of mantle remnants after high degree of partial melting. (A) The thermodynamic information recorded by a relatively oxidized depleted MORB mantle source ($Fe^{3+}/\Sigma Fe$=0.04) after undergoing 38% melt extraction at 4 GPa and 1750 °C. The star indicates the *P-T* conditions of melting. Under these conditions, the residual mantle exhibits a high Cr#, an oxygen fugacity of QFM-1.3, and a higher spinel $Fe^{3+}/\Sigma Fe$ ratio; (B) The thermodynamic information recorded by a relatively reduced depleted MORB mantle source ($Fe^{3+}/\Sigma Fe$=0.01) after undergoing 38% melt extraction at 4 GPa and 1750 °C. The star indicates the *P-T* conditions of melting. Under these conditions, the residual mantle exhibits a high Cr#, an oxygen fugacity of QFM-3.8, and a lower spinel $Fe^{3+}/\Sigma Fe$ ratio; (C) The thermodynamic information recorded by a relatively oxidized depleted MORB mantle source ($Fe^{3+}/\Sigma Fe$=0.04) after undergoing 26% melt extraction at 4 GPa and 1690 °C. The pentagon indicates the *P-T* conditions of melting. Under these conditions, the residual mantle exhibits a high Cr#, an oxygen fugacity of QFM-1.3, and a higher spinel $Fe^{3+}/\Sigma Fe$ ratio; (B) The thermodynamic information recorded by a relatively reduced depleted MORB mantle source ($Fe^{3+}/\Sigma Fe$=0.01) after undergoing 26% melt extraction at 4 GPa and 1690 °C. The pentagon indicates the *P-T* conditions of melting. Under these conditions, the residual mantle exhibits a high Cr#, an oxygen fugacity of QFM-3.8, and a lower spinel $Fe^{3+}/\Sigma Fe$ ratio.

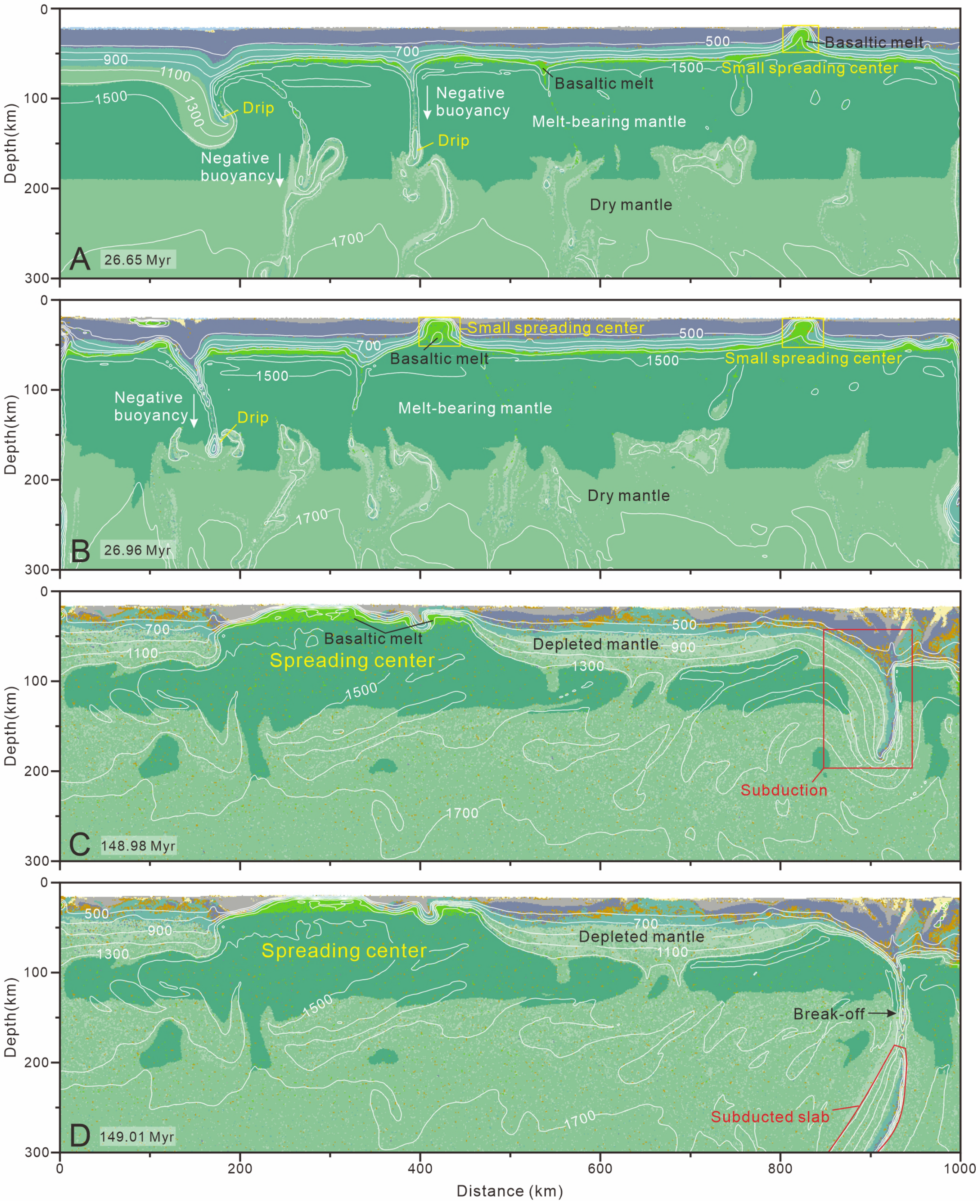

Fig. S6 Numerical simulation results of early Archean geodynamic evolution. (A-B) The early-Archean simulation indicates that the thick oceanic crust, composed of cold and dense material, experienced subsidence due to negative buoyancy[4,5]. This process triggered the formation of several mantle upwelling zones, where ascending, hotter, melt-bearing peridotite underwent additional decompression melting. This led to the generation of basaltic magma, which subsequently formed new hydrated basaltic lavas at the crust surface. The mantle became progressively depleted due to melt extraction. (C-D) A series of spreading centers formed, producing basaltic melts that generated a new basaltic crust. Over time, this process facilitated the formation of oceanic slabs. A large spreading center emerged, with weak zones developing along its margins, eventually giving rise to Archean subduction events characterized by frequent slab break-off.

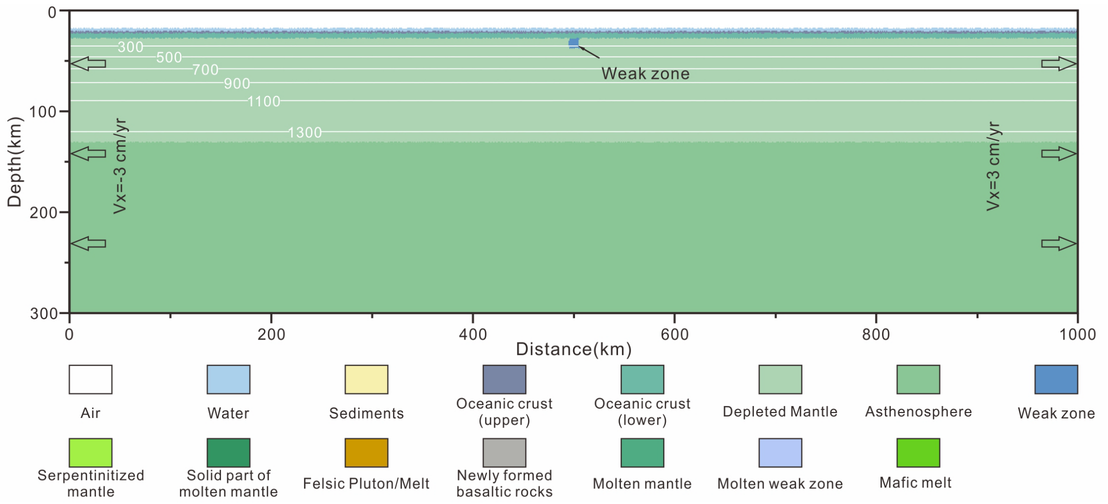

Fig. S7 Initial model of mid ocean ridge expansion experiment.

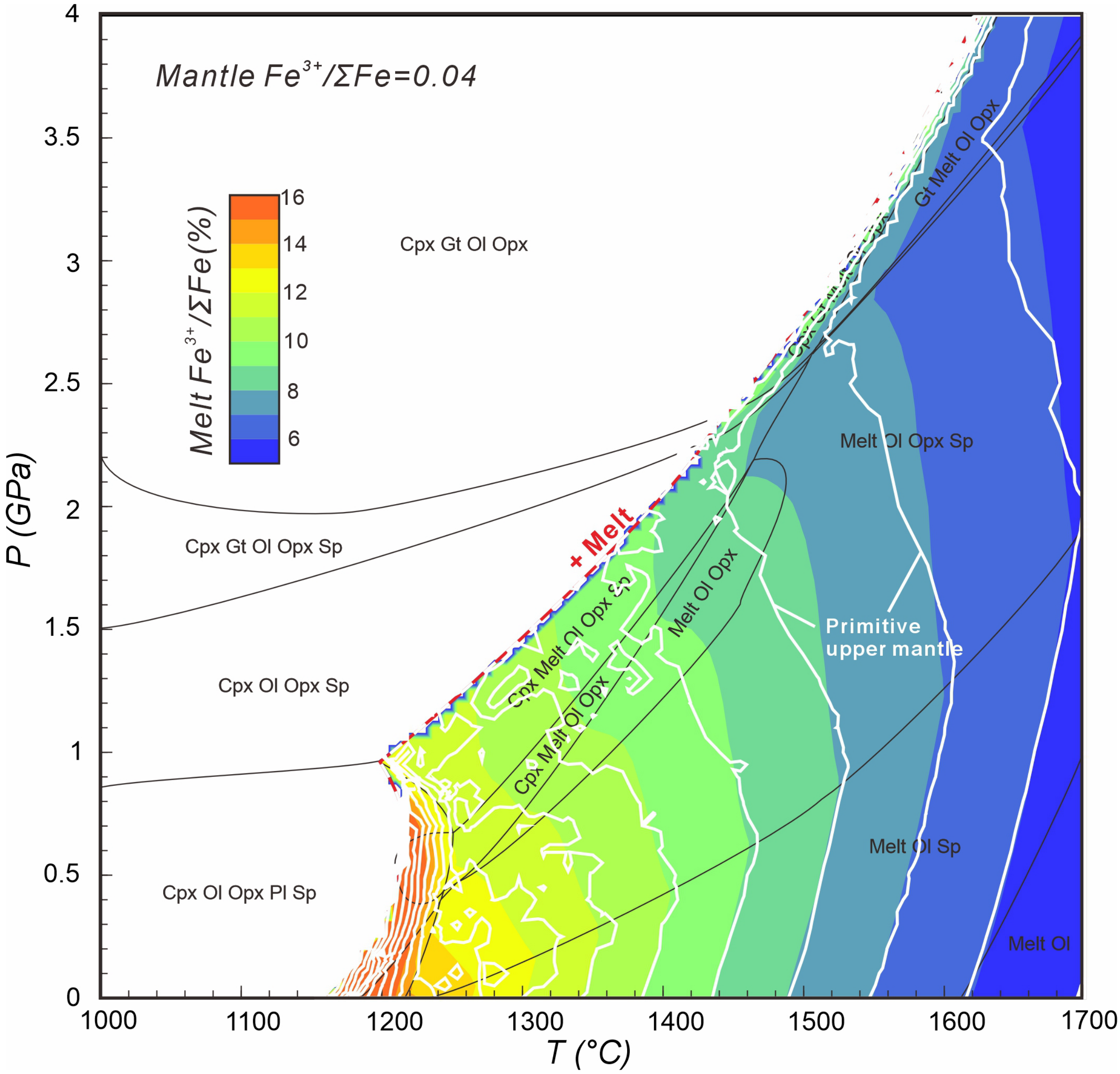

Fig. S8. Phase equilibrium modeling of a depleted MORB mantle source and a primitive mantle source (Supplementary Data 1). The bulk rock $Fe^{3+}/\Sigma Fe$ ratio is uniformly set to 0.04. The background black lines and colored fields represent mineral phase boundaries and melt $Fe^{3+}/\Sigma Fe$ ratios for the depleted MORB mantle source, consistent with Figure 1A. Superimposed white contour lines indicate $Fe^{3+}/\Sigma Fe$ values corresponding to the boundaries of the colored fields below. These results show that the compositional transition from the primitive upper mantle to the depleted MORB mantle has little effect on both the absolute values and the overall trend of the modeled melt $Fe^{3+}/\Sigma Fe$ ratio.

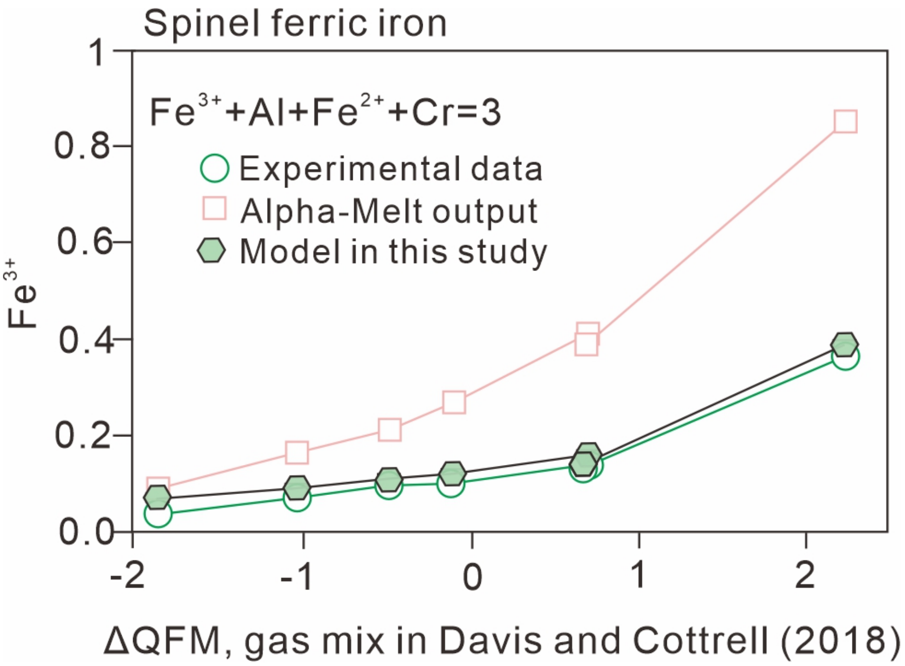

Fig. S9 The comparison between thermodynamic simulation and experimental petrology data[6] indicates that the simulation method in this study will not deviate due to the inappropriate Fe^{3+} behavior of spinel.

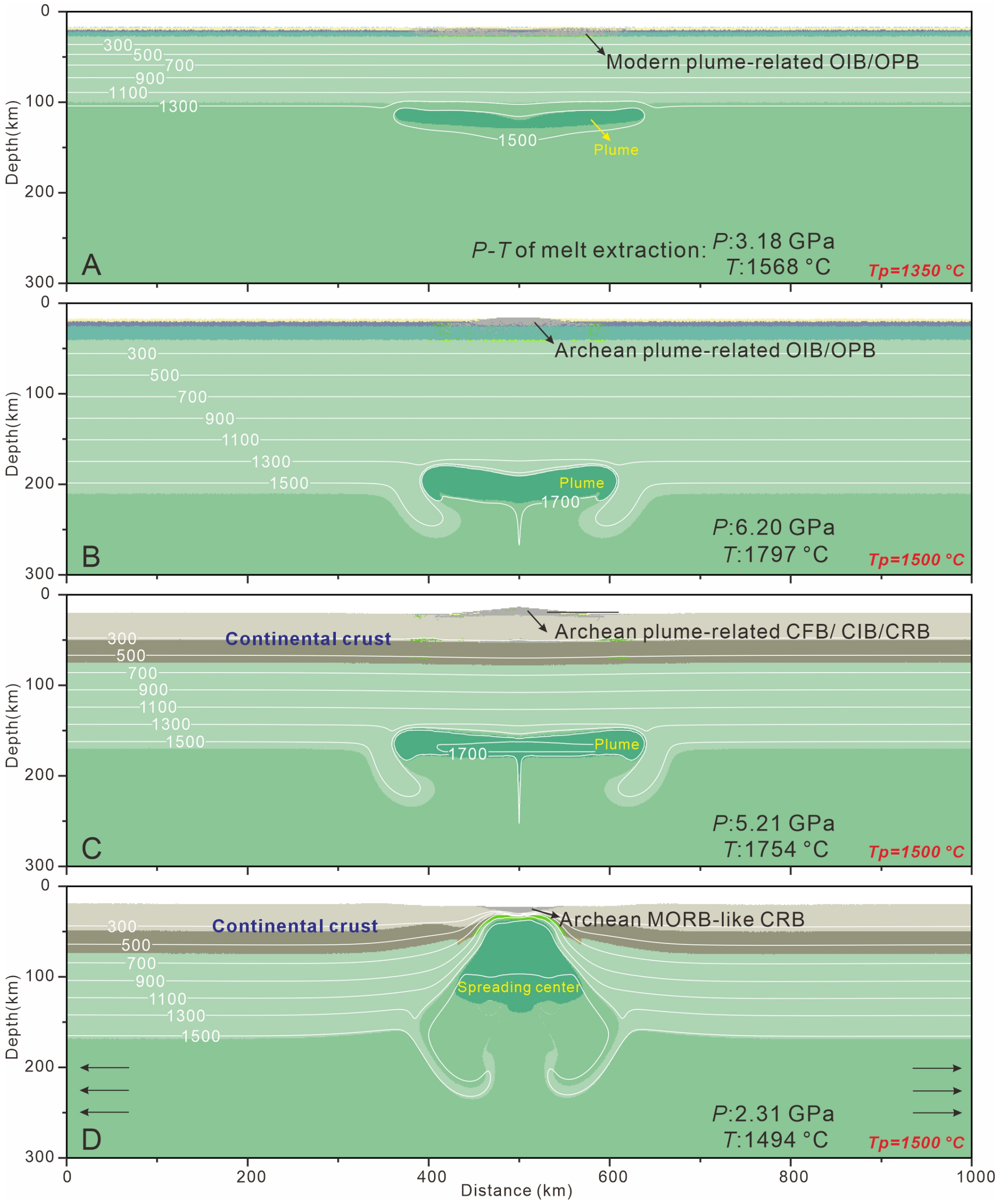

Fig. S10 Numerical modeling of average melt extraction *P-T* conditions under possible modern (1350 °C) and Archean (1500 °C) mantle potential temperatures indicates that melt extraction associated with mantle plumes occurs under significantly higher pressure-temperature conditions than those recorded in our database. (A) *P-T* conditions for melt extraction of ocean island basalts (OIB) and ocean plateau basalts (OPB) associated with mantle plumes under modern mantle potential temperatures; (B) *P-T* conditions for melt extraction of OIB/OPB under Archean mantle potential temperatures, where it is noteworthy that some melts may be komatiitic in nature; (C) *P-T* conditions for melt extraction of continental flood basalts (CFB), continental intraplate basalts (CIB), and continental rift basalts (CRB) associated with mantle plumes under Archean mantle potential temperatures, where some melts may also be komatiitic; (D) *P-T* conditions for melt extraction of extension-related (MORB-like) continental rift basalts (CRB) that may have existed under Archean mantle potential temperatures. The MORB-like CRB exhibits melt extraction conditions similar to those in oceanic spreading environments (Fig. 2).

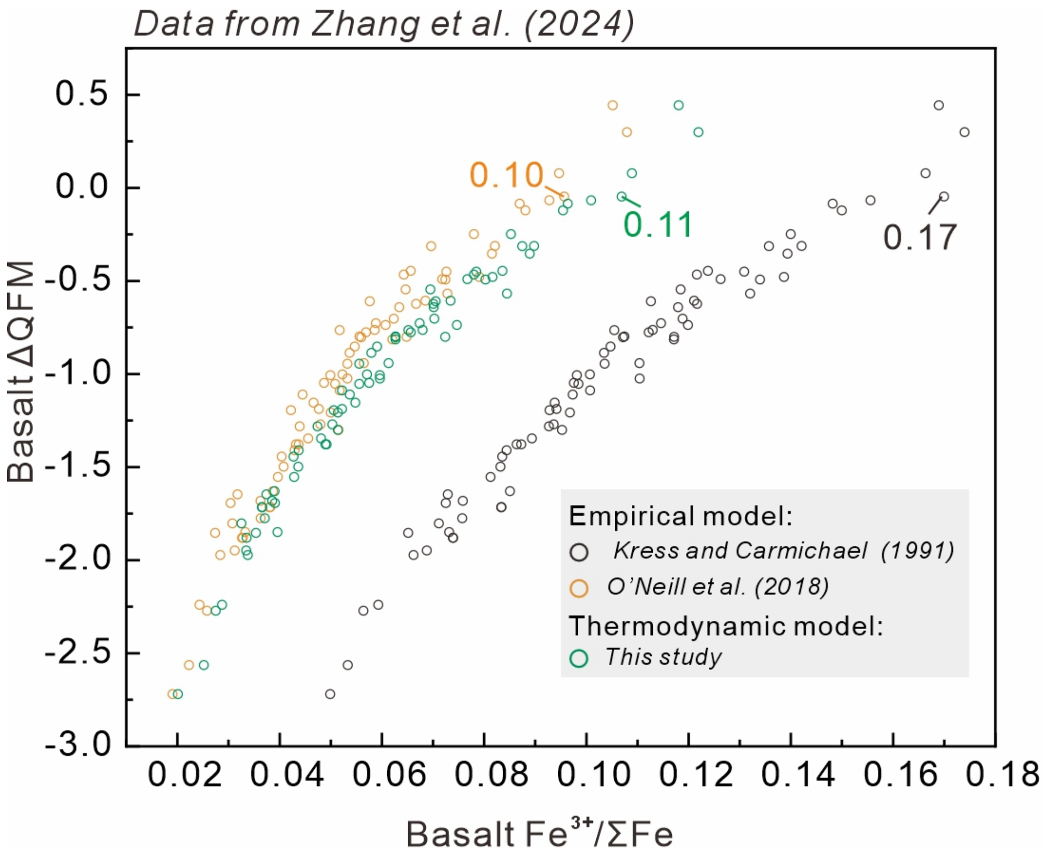

Fig. S11 The thermodynamic method used in this study and two empirical methods calculated the whole-rock $Fe^{3+}/\Sigma Fe$ results of basalt under the same *P-T-fO₂* conditions. The empirical results using the method of Kress and Carmichael[7] come from Zhang et al.[3]. The empirical results used method of O'Neill et al.[8] and the thermodynamic results are calculated by this study. These calculation results indicate that the method of Kress and Carmichael[7] significantly overestimates the whole rock $Fe^{3+}/\Sigma Fe$ of basalt.

References

1. Wang, J., Xiong, X., Takahashi, E., Zhang, L., Li, L. & Liu, X. Oxidation state of arc mantle revealed by partitioning of V, Sc, and Ti between mantle minerals and basaltic melts. J. Geophys. Res. Solid Earth 124, 4617–4638 (2019).

2. Gao, L., Liu, S., Cawood, P. A., Hu, F., Wang, J., Sun, G. & Hu, Y. Oxidation of Archean upper mantle caused by crustal recycling. *Nat. Commun.* **13**, 3283 (2022).

3. Zhang, F., Stagno, V., Zhang, L., Chen, C., Liu, H., Li, C. & Sun, W. The constant oxidation state of Earth's mantle since the Hadean. *Nat. Commun.* **15**, 6521 (2024).

4. Sizova, E., Gerya, T., Stüwe, K. & Brown, M. Generation of felsic crust in the Archean: a geodynamic modeling perspective. *Precambrian Res.* **271**, 198–224 (2015).

5. Sizova, E., Gerya, T., Brown, M. & Stüwe, K. What drives metamorphism in early Archean greenstone belts? Insights from numerical modeling. *Tectonophysics* **746**, 587–601 (2018).

6. Davis, F. A. & Cottrell, E. Experimental investigation of basalt and peridotite oxybarometers: Implications for spinel thermodynamic models and Fe^{3+} compatibility during generation of upper mantle melts. *Am. Mineral.* **103**, 1056–1067 (2018).

7. Kress, V. C. & Carmichael, I. S. E. The compressibility of silicate liquids containing Fe_2O_3 and the effect of composition, temperature, oxygen fugacity and pressure on their redox states. *Contrib. Mineral. Petrol.* **108**, 82–92 (1991).

8. O'Neill, H. St. C., Berry, A. J. & Mallmann, G. The oxidation state of iron in mid-ocean ridge basaltic (MORB) glasses: implications for their petrogenesis and oxygen fugacities. *Earth Planet. Sci. Lett.* **504**, 152–162 (2018).
