# Supplementary material for: The Mantle Fe3+/ΣFe Ratio Has Doubled Since the Early Archean
Source: Nat Commun. 2026 Jan 14;17:429. doi: 10.1038/s41467-025-66969-1 (PMC12804952; doi:10.1038/s41467-025-66969-1)
Supplement: Supplementary file 2 — Description of Additional Supplementary Information [file 41467_2025_66969_MOESM2_ESM.pdf]

Description of Additional Supplementary Information

Supplementary Data 1. Initial mantle whole-rock composition in modeling (wt%).

Supplementary Data 2. Different test conditions and the corresponding results in numerical experiments.

Supplementary Data 3. The rock geochemistry database used in this study.

Supplementary Data 4. Physical properties of rocks used in numerical experiments.
